# Cutaneous Extramedullary Hematopoiesis in Primary Myelofibrosis: A Case Report With Rapidly Fatal Outcome

**DOI:** 10.7759/cureus.103957

**Published:** 2026-02-20

**Authors:** Assia El Bouhmadi, Bouchra Baghad, Hanane Rachadi, Fatima Zahra Benhayoun, Soumiya Chiheb

**Affiliations:** 1 Dermatology, Ibn Rochd University Hospital Center, Faculty of Medicine and Pharmacy, Hassan II University of Casablanca, Casablanca, MAR

**Keywords:** dermatological manifestation, extramedullary hematopoiesis, failure of the bone marrow, jak2 mutation, myelofibrosis

## Abstract

Extramedullary hematopoiesis (EMH) is the formation of hematopoietic cells outside the bone marrow, typically as a compensatory mechanism in the setting of marrow failure. Cutaneous EMH is an exceptionally rare manifestation, usually associated with chronic myeloproliferative disorders such as primary myelofibrosis. We report the case of a 54-year-old woman with Janus kinase 2 (JAK2)-positive primary myelofibrosis and long-standing transfusion-dependent cytopenias who presented with multiple firm, erythematous-to-violaceous nodular lesions on the trunk. Histological examination of a skin biopsy revealed a dermal infiltrate composed of myeloid precursors with diffuse myeloperoxidase expression, consistent with cutaneous EMH. Bone marrow evaluation confirmed advanced myelofibrosis without acute transformation. Despite supportive care, the patient's condition rapidly deteriorated, and she died two weeks after presentation. This case highlights the rarity of cutaneous EMH, its association with advanced marrow failure, and its poor prognostic significance. Recognition of this manifestation may provide insight into the severity of hematopoietic exhaustion and guide clinical management in patients with myeloproliferative disorders.

## Introduction

Extramedullary hematopoiesis (EMH) is defined as the production of one or more hematopoietic cell lineages outside the bone marrow [1]. It represents a compensatory response to ineffective medullary hematopoiesis, leading to the development of hematopoietic tissue in non-medullary sites. EMH most commonly involves organs with embryonic hematopoietic activity, such as the liver and spleen, but may also occur in atypical locations including the lungs, kidneys, and gastrointestinal tract [2].

Primary myelofibrosis is a chronic myeloproliferative neoplasm characterized by clonal stem cell proliferation, progressive bone marrow fibrosis, and ineffective hematopoiesis. Activating mutations in the Janus kinase-signal transducers and activators of transcription (JAK-STAT) pathway, particularly JAK2 V617F, play a central role in disease pathogenesis. Progressive marrow failure promotes the mobilization of hematopoietic progenitor cells and their homing to extramedullary sites, predisposing to EMH [3].

Cutaneous findings in myelofibrosis are usually nonspecific and related to cytopenias, such as pallor, petechiae, or ecchymoses. In contrast, true cutaneous EMH is exceedingly rare and may present diagnostic challenges, and its prognostic significance remains uncertain.

We report a rare case of cutaneous EMH in a 54-year-old woman with JAK2-positive primary myelofibrosis, associated with a rapidly fatal outcome.

## Case presentation

A 54-year-old woman with a two-year history of JAK2 V617F-positive primary myelofibrosis was referred for the recent onset of cutaneous lesions. Since diagnosis, the disease course had been characterized by progressive bone marrow failure requiring regular red blood cell and platelet transfusions. At the time of dermatologic evaluation, laboratory testing showed severe anemia (hemoglobin 6.5 g/dL; reference range 12-16 g/dL) and thrombocytopenia (platelets 38 × 10⁹/L; reference range 150-400 × 10⁹/L), with ongoing transfusion dependency. No morphologic or clinical evidence of leukemic transformation had been documented during follow-up.

**Figure 1:**
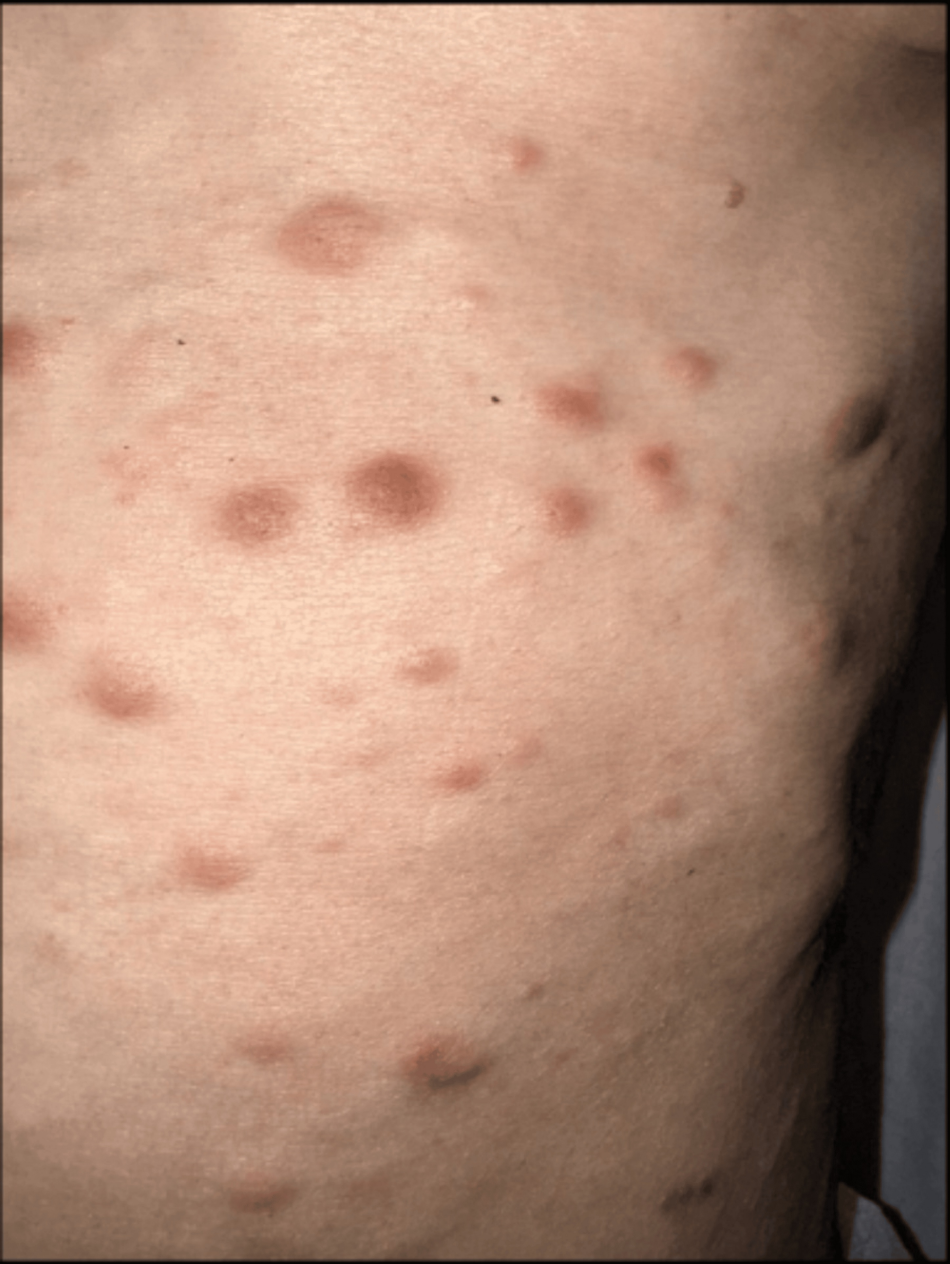
Close-up view of the trunk showing multiple erythematous-to-violaceous papules and nodules. The lesions are firm, infiltrated, non-ulcerated, and non-scaly. Lesion size ranges from a few millimeters to approximately 2 cm. The absence of surface change, epidermal disruption, or hemorrhagic crusts supports a primarily dermal infiltrative process.

**Figure 2:**
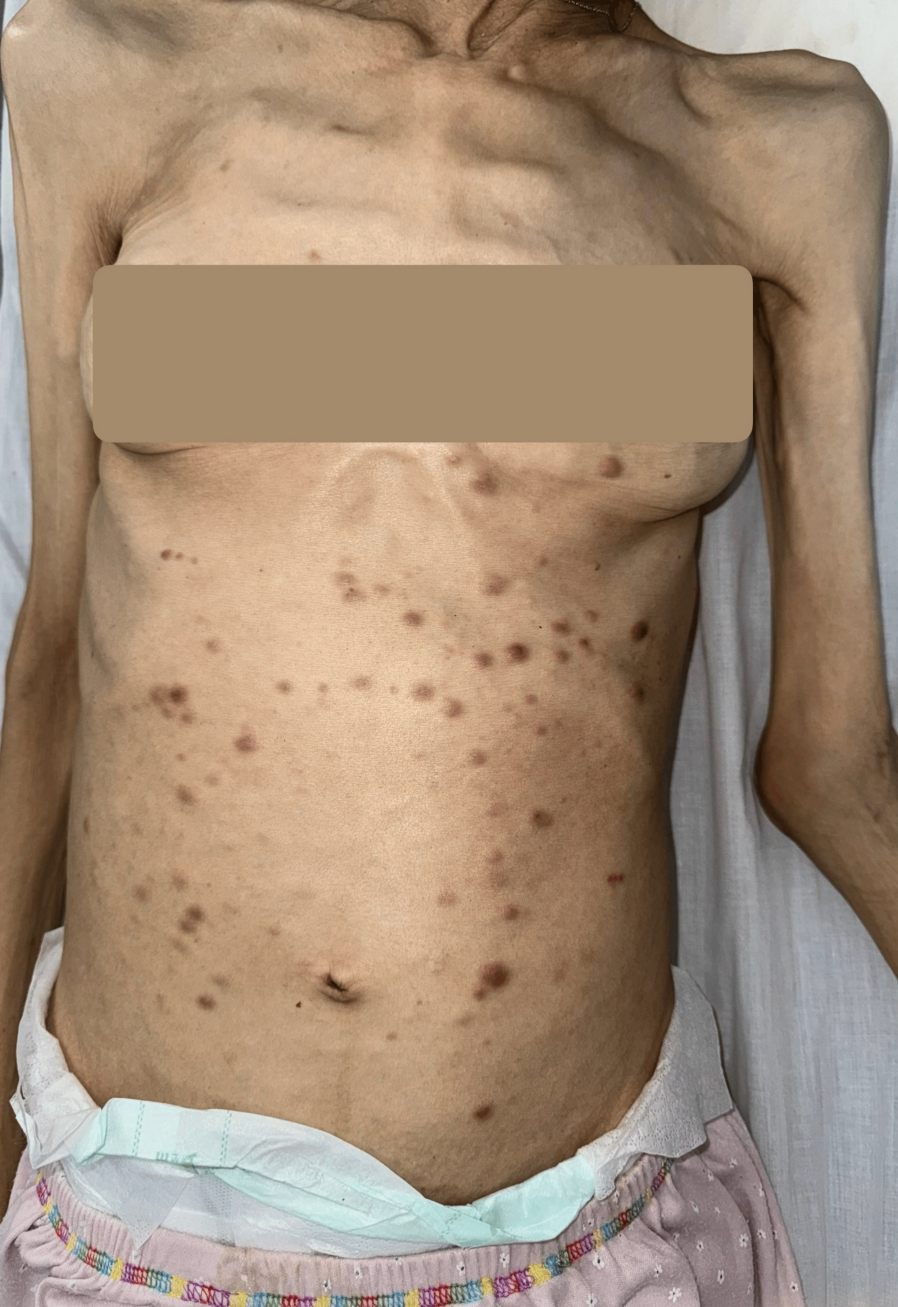
General clinical view of the patient's trunk demonstrating the widespread distribution of multiple papulonodular lesions predominantly involving the anterior trunk, with relative sparing of the face and extremities. Marked cachexia and severe weight loss are evident, reflecting advanced systemic disease.

Cutaneous lesions had appeared approximately one month prior to consultation and had progressively increased in number. Physical examination revealed multiple firm, infiltrated erythematous-to-violaceous papules and nodules measuring approximately 0.5-2 cm in diameter (Figure 1), predominantly distributed on the anterior trunk (Figure 2). The lesions were non-ulcerated, non-pruritic, and painless. No mucosal involvement was observed. There were no associated signs of infection, hemorrhage, or necrosis. The patient had a known massive splenomegaly related to her underlying hematologic disease. Her general condition was severely altered, with marked asthenia, significant weight loss, and poor functional status.

Histopathological examination of a 6 × 4 mm punch biopsy revealed a dense, diffuse dermal infiltrate with sparing of the epidermis. The infiltrate consisted of a polymorphic population of hematopoietic cells, including numerous granular cells at different stages of maturation. Immunohistochemical analysis demonstrated diffuse cytoplasmic positivity for myeloperoxidase (MPO), supporting myeloid lineage differentiation and consistent with cutaneous EMH. No epidermotropism, blast-rich population, or cohesive tumoral architecture suggestive of myeloid sarcoma was identified.

A concomitant bone marrow biopsy confirmed advanced-stage primary myelofibrosis without morphologic evidence of acute leukemic transformation. Despite continued supportive transfusion therapy, hematologic parameters remained poor. The patient's clinical condition deteriorated rapidly, and she died two weeks after dermatologic evaluation from multiorgan failure related to advanced bone marrow failure and disease progression.

## Discussion

Cutaneous EMH represents an exceptionally rare manifestation, most often described in the setting of chronic myeloproliferative neoplasms, particularly primary myelofibrosis, and more rarely in congenital hemolytic anemias or myelodysplastic syndromes [4]. First reported by Hickling in 1937 [5], cutaneous EMH typically presents as firm erythematous-to-violaceous papules or nodules, predominantly involving the trunk, and may clinically mimic cutaneous metastases, leukemia cutis, or inflammatory dermatoses. To date, only a limited number of cases have been documented in the literature, underscoring the rarity of this entity.

In the present case, cutaneous EMH occurred in a patient with JAK2 V617F-positive primary myelofibrosis complicated by profound anemia, severe thrombocytopenia, transfusion dependence, and massive splenomegaly. These features reflect advanced bone marrow failure, a recognized context favoring the mobilization of hematopoietic progenitor cells and the development of EMH. The relatively rapid onset and progression of cutaneous lesions further suggest a late-stage manifestation of systemic disease.

Histologically, cutaneous EMH is classically characterized by a polymorphous dermal infiltrate composed of hematopoietic precursors from one or more lineages, including myeloid, erythroid, and megakaryocytic elements. However, as highlighted in the review by Mizoguchi et al., many reported cases demonstrate involvement of only one or two lineages rather than true trilineage hematopoiesis [6]. Consequently, immunohistochemistry plays a central role in confirming the diagnosis and excluding major differential diagnoses such as leukemia cutis or myeloid sarcoma. Commonly recommended markers include MPO for myeloid differentiation, glycophorin A or spectrin for erythroid lineage, and CD61 for megakaryocytes [7].

In our patient, diffuse MPO expression confirmed the presence of a myeloid hematopoietic component, supporting the diagnosis of cutaneous EMH. The absence of demonstrable erythroid or megakaryocytic precursors may reflect either true lineage restriction or, more likely, technical limitations related to the unavailability of lineage-specific immunohistochemical markers such as CD61 and spectrin in our laboratory. Similar limitations have been reported in previous case series and may lead to the underrecognition of multilineage hematopoiesis in cutaneous lesions [7].

From a prognostic perspective, cutaneous involvement by EMH is generally considered a marker of advanced disease and has been associated with poor outcomes. Koch et al. reported a median survival of approximately 13 months in patients with non-hepatosplenic EMH, compared with nearly five years in typical primary myelofibrosis, while earlier reports described survival ranging from two to seven months following the appearance of cutaneous lesions [8]. In our case, the rapidly fatal course, with death occurring only two weeks after dermatologic presentation, further supports the association between cutaneous EMH and advanced, terminal-stage myelofibrosis.

## Conclusions

In this case, the coexistence of transfusion-dependent cytopenia and cutaneous EMH highlights a rare manifestation of advanced primary myelofibrosis. While the temporal association suggests a possible link with disease progression, the limited single-case data do not allow firm conclusions regarding prognostic significance. Evaluating the extent and localization of EMH, particularly cutaneous involvement, may provide useful insights for patient monitoring and multidisciplinary management. Awareness of this unusual presentation is important for dermatologists, as skin lesions may occasionally be among the first visible signs of systemic hematologic disease. Further reports and studies are needed to clarify the clinical implications of cutaneous EMH in myeloproliferative disorders.
